# Acute interstitial nephritis after messenger RNA-based vaccination

**DOI:** 10.1093/ckj/sfab180

**Published:** 2021-09-28

**Authors:** Cecilia Czerlau, Federica Bocchi, Charalampos Saganas, Bruno Vogt

**Affiliations:** Department for Nephrology and Hypertension, University Hospital Insel Bern, Bern, Switzerland; Department for Nephrology and Hypertension, University Hospital Insel Bern, Bern, Switzerland; Department for Pathology, University Hospital Insel Bern, Bern, Switzerland; Department for Nephrology and Hypertension, University Hospital Insel Bern, Bern, Switzerland

**FIGURE 1:**
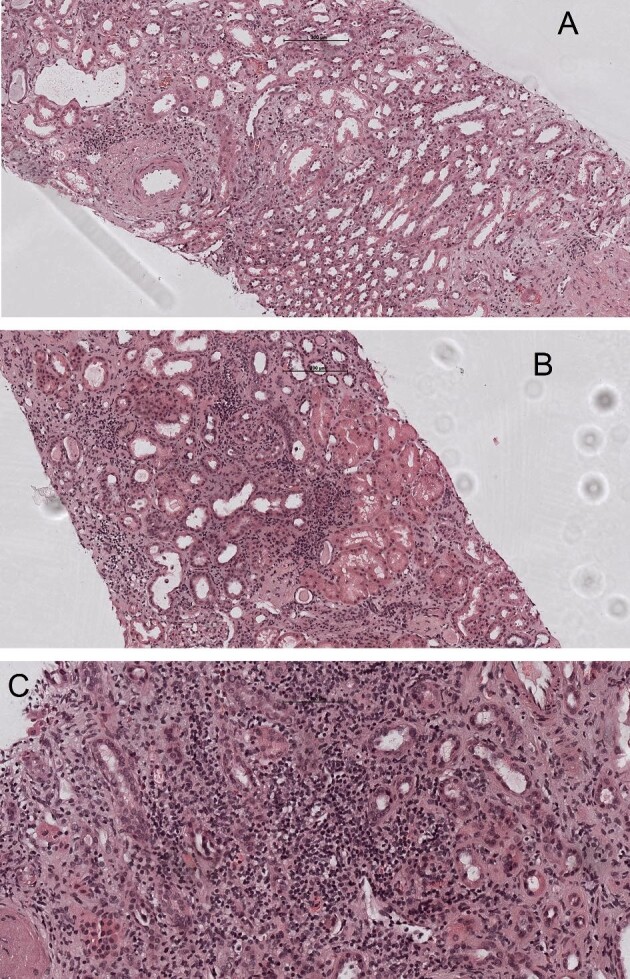
(A–C) Acute tubulointerstitial nephritis with tubulitis (haematoxylin and eosin stain) and interstitial oedema (haematoxylin and eosin stain, 10×).

Severe acute respiratory syndrome-2 virus (SARS-CoV-2) is responsible for the current global coronavirus disease 2019 (COVID-19) pandemic [1]. The messenger RNA (mRNA)-based vaccines positively changed the course of the SARS-CoV-2 pandemic. Although both vaccines [mRNA-1273 (Moderna) and BNT162b2 (Pfizer-BioNTech)] have shown great efficacy and have decreased the rate of infection, there is still uncertainty about their side effects [2]. In addition to transient local and systemic side effects, recently isolated cases of renal damage in relation to vaccination have been reported. In the last few months, de novo vasculitis, cases of minimal change disease (MCD) and occasional recurrence of primary disease have been described after mRNA-based vaccine [3]. We report five cases with an unusual picture of acute interstitial nephritis (AIN; histologically mainly lymphocytes, plasma cells and macrophages, as well isolated neutrophils and eosinophils; Figure 1) shortly after receiving mRNA-based vaccine. The key patient characteristics are shown in Table 1.

**Table 1.** Key patient characteristics

Characteristics	Patient 1	Patient 2	Patient 3	Patient 4	Patient 5
Pre-existing nephropathy	No	No	Yes, FSGS	No	No
Kidney values before vaccination	76.5 μmol/L	No previous values available	167 μmol/L	76 μmol/L	49 μmol/L
Kidney values after vaccination	355 μmol/L	268 μmol/L	355 μmol/L	86 μmol/L	100 μmol/L
Urinalysis					
Proteinuria	8.3 g/L	9.7 g/L	3.2 g/L	0.61 g/L	2 g/L
Albuminuria	1251 g/molCr	994 g/molCr	886 g/mmolCr	26 g/molCr	Unclear
Glomerular erythrocytes	4%	25%	No	56%	12%
Nephrotic syndrome	No	Yes	Yes	No	No
Immunoserology	Unremarkable	Unremarkable	Unremarkable	Unremarkable	Unremarkable
Kidney biopsy	Lymphocytes, plasma cells, macrophages, eosinophilic granulocytes, and some neutrophilic granulocytes, tubulitis and interstitial oedema	Lymphocytes, plasma cells, macrophages, and eosinophilic granulocytes, two granulomas, tubulitis, and tubule destruction	Lymphocytes, plasma cells, macrophages, and sporadic neutrophilic granulocytes with tubulitis and interstitial oedema	Lymphocytes, plasma cells, macrophages, sporadic eosinophilic granulocytes and neutrophil granulocytes with tubulitis and interstitial oedema	Lymphocytes, plasma cells, macrophages, sporadic eosinophilic granulocytes and neutrophil granulocytes with tubulitis and interstitial oedema
	EM: no evidence of minimal change disease	FSGS with 2 segmental scleroses	EM: fusion of podocyte extensions, narrow basement membrane. No deposits	EM: mesangial IgA deposition	EM: mesangial IgA deposition
		EM: fusion of podocyte extensions, 2 segmental scleroses and prominent ischaemoid changes			
Cortisone therapy	Yes	Yes	Yes	Yes	Yes
Kidney values after treatment	88 μmol/L	235 μmol/L	210 μmol/L	72 μmol/L	90 μmol/L

Cr, creatinine; EM, electron microscopic examination.

Patient 1 was a 55-year-old man with a history of hypertension and prostate cancer treated with prostatectomy. He developed, 4 days after the second dose of Pfizer-BioNTech vaccine, sudden bilateral renal pain. Laboratory testing revealed acute kidney injury (AKI), inactive urine sediment and heavy proteinuria (11 g/d, 1251 g/mmol creatinine albuminuria) without oedema, hypoalbuminemia or hypercholesterolemia. Renal biopsy showed AIN with acute tubular involvement and the electron microscopy showed no evidence of MCD. Renal function and proteinuria were determined 1 week before the second vaccination during urological care and were unremarkable.

Patient 2 was a 54-year-old man, known for a history of myocardial infarction, who presented with glomerular erythrocyturia and nephrotic syndrome 3 days after the second dose of Moderna vaccine. The kidney biopsy showed AIN with granulomas and tubular destruction. Subsequent electron microscopic examination revealed evidence of focal segmental glomerulosclerosis (FSGS) with fusion of podocyte extensions, two segmental scleroses and prominent ischemoid changes. Because the patient was not under regular medical care and had no complaints, it remains unknown whether he had pre-existing FSGS and additionally developed interstitial nephritis after SARS-CoV-2 vaccination or whether both entities occurred after vaccination.

Patient 3 was a 58-year-old man with primary FSGS diagnosed in 2013 and refractory to treatment with several recurrences in the past. The patient reported increased oedema a few days after the second dose of Moderna vaccine. Laboratory examination revealed AKI with a picture of nephrotic syndrome. A renal biopsy revealed extensive AIN. Focal and segmental involvement with sclerosis in 5 of 25 glomeruli was also described, with no evidence of disease recurrence. Electron microscopic examination revealed a high degree of fusion of podocyte extensions, consistent with the known FSGS.

Patient 4 was a 38-year-old woman who was under regular medical care due to ulcerative colitis. One month after the second dose of Moderna vaccine, a routine examination revealed active urine sediment and proteinuria of 0.5 g/day. Renal biopsy showed AIN and immunofluorescence demonstrated mesangial immunoglobulin A (IgA) deposits. This was confirmed by electron microscopy. The cause of the interstitial nephritis may be related to chronic inflammatory bowel disease. However, the bowel disease was well controlled with ustekinumab for several years before the SARS-CoV-2 vaccination. Based on the temporal correlation, and with well-controlled underlying disease, vaccine-induced AIN is most likely.

Patient 5 was a 35-year-old woman who has been taking certolizumab since 2016 for rheumatoid arthritis. At the end of March she received the second dose of Pfizer-BioNTech vaccine, after which she felt unwell and tired. Laboratory chemistry showed a 50% increase in creatinine compared with the previous values from last year. Renal biopsy showed evidence of IgA nephropathy with crescents and hypercellularity as well as AIN. Interstitial nephritis may occur in the setting of IgA nephropathy, but it is usually less severe. It remains unclear whether the interstitial nephritis occurred in the setting of certolizumab therapy or after vaccination. The temporal correlation suggests vaccination-caused interstitial nephritis.

To the best of our knowledge, these are the first described cases of AIN after vaccination with an mRNA-based vaccine. AIN is an inflammatory pathology of renal interstitium and tubules that occurs on an allergic basis with oedema and acute damage and can potentially heal with interstitial fibrosis [4]. The exact mechanism of allergic reactions associated with mRNA vaccines is unclear. The systematic review and meta-analysis by Chen *et al.* [5] found that the incidence of anaphylaxis associated with the Pfizer-BioNTech vaccine was approximately 10 times higher than that associated with all previous vaccines. One possible explanation is that some individuals are at higher risk for non-IgE-mediated mast cell activation or complement activation. This is related to the lipid or polyethylene glycol lipid component of the vaccine. Early evaluation for AIN in patients with pre-existing renal disease or without pre-existing nephropathy is warranted if renal function worsens after vaccination with mRNA-based SARS-CoV-2 vaccines. In some cases it is not easy to differentiate whether worsening renal function is a recurrence of the previously known nephropathy (patient 3), a previously undiagnosed underlying disease (eventually patient 2) or a new disease. Steroid therapy was used in all patients with good effect, except patient 2, and might be the treatment of choice.
